# Chances and challenges in China

**DOI:** 10.1007/s13238-015-0235-4

**Published:** 2015-12-19

**Authors:** Boris Tefsen

**Affiliations:** Department of Biological Sciences, Xi’an Jiaotong-Liverpool University, Suzhou, China

So there I was, 35 years old and at the crossroads of my scientific career. The current academic system in the Netherlands, like in many other places around the world (Powell, 2015), is not designed to have scientists staying in a postdoc position forever. So, you are either deemed good enough to become a permanent staff member, or you have to leave academia at some point and try your luck in a pharmaceutical company or elsewhere. I had not succeeded in the former, but I also did not want to do the latter, so I took a less threaded path and headed to China.

**Figure 1:**
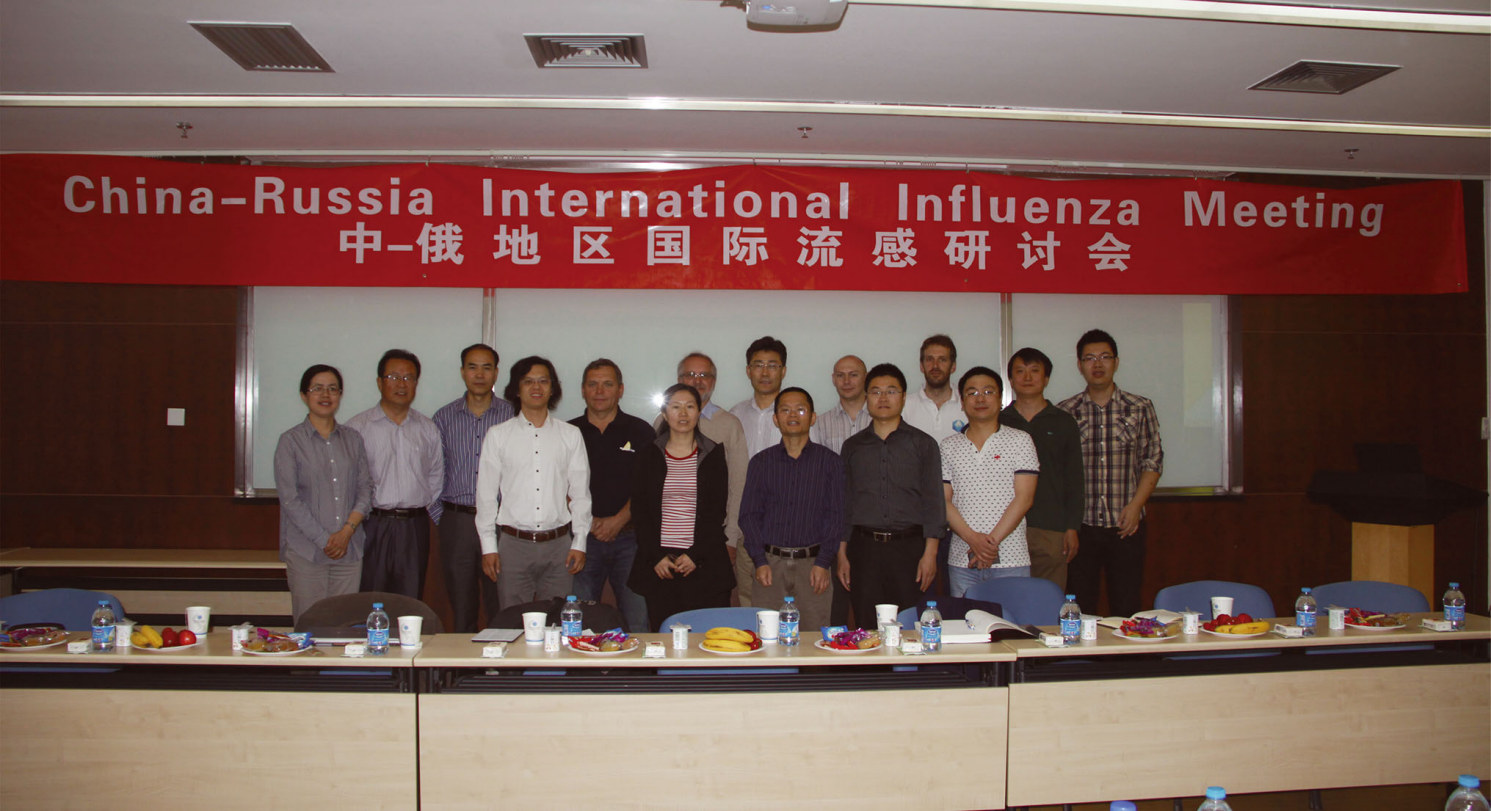
A joint meeting between China and Russia on influenza research held at the Institute of Microbiology, Chinese Academy of Sciences in Beijing in 2014

China seemed a good place to take my chances, as it is one of the few countries where funding for R&D is growing instead of shrinking (Van Noorden, 2014). I was lucky to get in touch with Professor George F. Gao in Beijing, who advised me to apply for a Fellowship for International Young Scientists and come work in his Structural Virology lab. This type of fellowship is one of the many funded by the Chinese Academy of Sciences with the aim to enhance the influx of foreign scientists into its institutes (Fig. 1).

After having received the positive outcome of my application and arranging the necessary paperwork to be able to work in China, my wife (who had found herself a job as well) and I left for Beijing in July 2012. Before arriving at the Institute of Microbiology (IMCAS), I had been exchanging emails with Christopher Vavricka, an American postdoc who was working in the same lab since 2009 (Vavricka, 2011). Although the information provided by him helped me to shape my expectations, the culture shock was still considerable. Fortunately, my Chinese colleagues were also very helpful and after a few weeks, I could already conduct my first experiments on one of the internal proteins of an influenza-like virus discovered recently in bats (Tefsen et al., 2014; Wu et al., 2014). During my period at the IMCAS, I witnessed the hard work and dedication of the students and staff in the lab. I also got a real taste of Chinese culture, as many events in Chinese life are accompanied by very delicious food.

In retrospect, I could not have come to a better laboratory, as the first cases of human-infecting H7N9 influenza strains made their appearance in China in the spring of 2013. Not only is influenza one of the main research topics in the lab of Gao, but he also serves as the Deputy Director-General of the Chinese Center for Disease Control and Prevention and therefore has the responsibility to supervise the battles against epidemics. During this hectic period, many of my colleagues were working around the clock to solve the structures of the influenza surface molecules hemagglutinin and neuraminidase identified from the different clinical H7N9 isolates (Shi et al., 2013; Wu et al., 2013) and to track down the origins of the virus (Liu et al., 2013).

Luckily, the H7N9 epidemic was relatively small and quickly under control, helped by adequate sharing of information and measures taken by the Chinese government, which had learned from the SARS epidemic that affected the country from 2002 to 2003. For me as a Dutchman working in a Chinese lab, an interesting by-product of a letter in Science about closing down live poultry markets to prevent new infections by H7N9 (Gao, 2014), was that Gao was mentioned in a Dutch national newspaper in a column by a prominent emeritus professor (Borst, 2014).

In the meantime, other viral threats emerged around the globe. The SARS-related corona virus MERS was causing several fatalities in the Middle East and it posed new challenges for the eager scientists in the Gao lab to solve. One extraordinary result was the elucidation of the complex structure of viral surface spike protein together with its CD26 receptor by Guangwen Lu and several other colleagues (Lu et al., 2013). In March 2014, another, far more destructive epidemic started.

**Figure 2:**
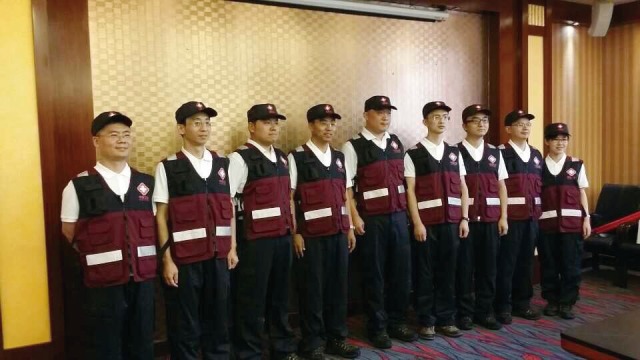
Jun Liu (third from right) and other members of the CDC-teams that went to Africa to help combat the Ebola outbreak in 2014

The Ebola virus struck Liberia, Sierra Leone and Guinea with great force and claimed many lives (Alexander et al., 2015). International aid was (and is) desperately needed in these countries and China offered help by sending medical teams to the stricken countries. The CDC-team going to Sierra Leone in 2014 was led by Gao for two months (Gao and Feng, 2014). One of my other colleagues, Jun Liu (Fig. 2), first went to Guinea and a few months later he went to help in Sierra Leone as well (Tong et al., 2015).

**Figure 3:**
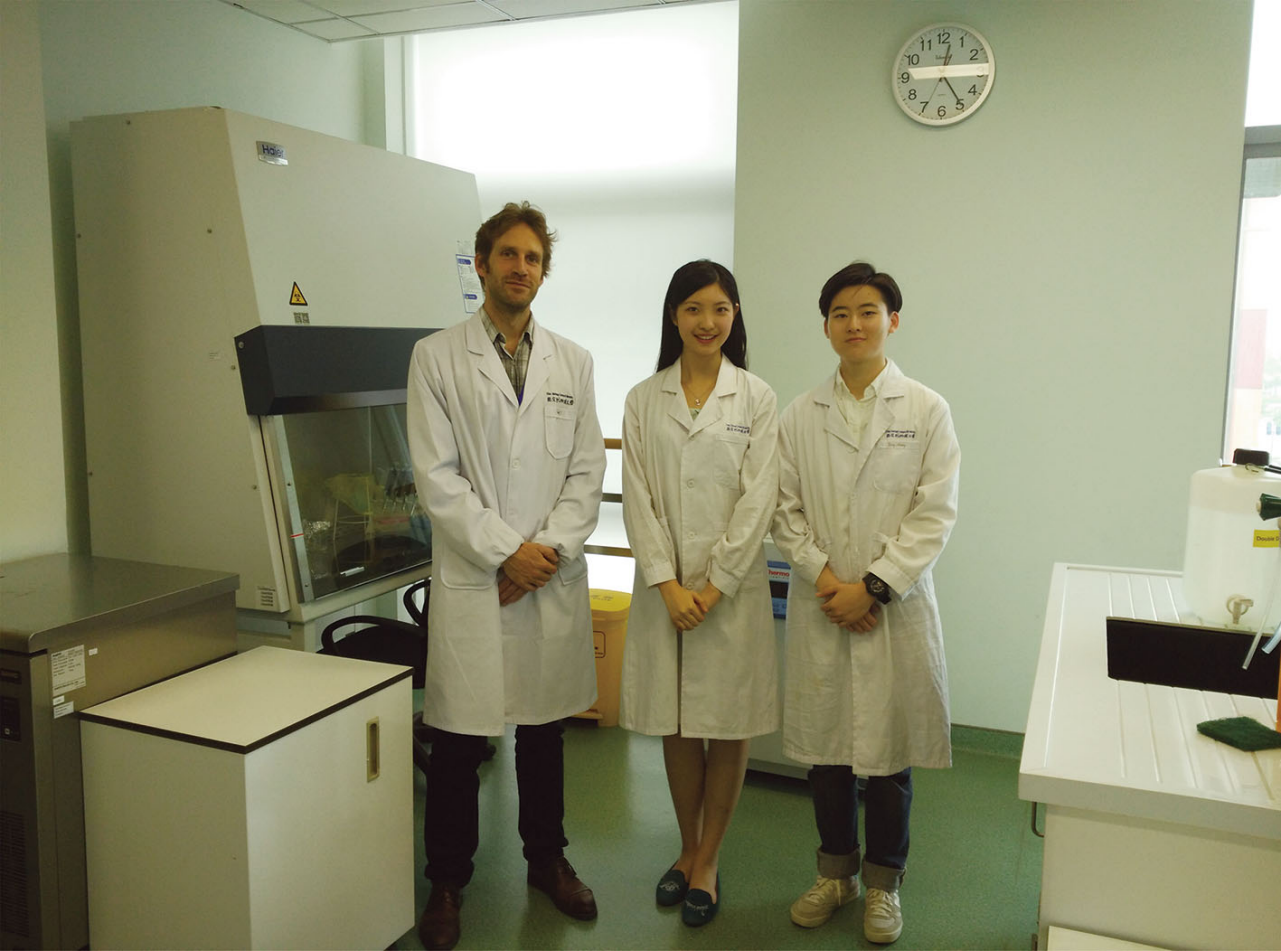
Posing in my lab at XJTLU together with two students, Xinzhu Fei (middle) and Jing Zhang (right)

Amidst all this global turmoil, I was about to leave Beijing to become an Associate Professor at the Xi’an Jiaotong-Liverpool University (XJTLU) in Suzhou, an ancient city close to Shanghai. This young university was founded in 2006 in a joint effort by Xi’an Jiaotong University, one of the C9 League universities in China, and the University of Liverpool and it aims to blend the best of both education systems into its own and be a world class research-led university. It offered me the chance to build up my own research lab and it seemed like everything had fallen into place for me (Fig. 3).

After one and a half year in Suzhou, I can only confirm my initial feelings. I enjoy teaching the eager students at my university, although the language—the curriculum is taught in English—poses sometimes difficulties to some of them during assessments. XJTLU is one of the experiments by the Chinese government to test education styles that differ from the classic Chinese education system and one of the main goals is to improve creative thinking amongst its student population. It is wonderful to see how our students are capable of detaching themselves quickly from twelve years of merely exam-oriented learning.

Setting up my own laboratory has also been very stimulating and I thoroughly enjoy the freedom of finally being able to completely follow my own research interests. My research focuses on the synthesis of the mycobacterial cell envelope and will hopefully contribute to combating the growing number of cases of patients that are infected with multi-drug resistant *Mycobacterium tuberculosis* (WHO, 2014). Three and a half years after going to China, I can only conclude that it worked out great for me and I am excited to see what the future will bring.
